# The miR-30c-5p/SOCS3 axis is a potential driver of inflammation and metabolic imbalance in Duchenne muscular dystrophy

**DOI:** 10.3389/fcell.2026.1841851

**Published:** 2026-05-22

**Authors:** Yayu Wang, Xiuyi Ai, Yue Chang, Shu Zhang, Pei Zhang, Shiwen Wu

**Affiliations:** 1 School of Clinical Medicine, Qinghai University, Xining, Qinghai, China; 2 Department of Neurology, First Medical Center of Chinese PLA General Hospital, Beijing, China; 3 Center for Translational Genomics and Rare Diseases, First Medical Center of Chinese PLA General Hospital, Beijing, China; 4 Beijing Key Laboratory of Gene Editing Therapy for Hereditary Neuromuscular Diseases, Beijing, China

**Keywords:** Duchenne muscular dystrophy, inflammation, metabolic dysfunction, miR-30c-5p, single-cell RNA sequencing, SOCS3

## Abstract

**Background:**

Duchenne muscular dystrophy (DMD) constitutes a severe, incurable disorder inherited in an X-linked manner, characterized by continuous skeletal muscle degeneration, chronic inflammatory responses, and profound metabolic alterations. Although miRNA-mRNA regulatory networks are thought to contribute to DMD pathogenesis, their key drivers remain insufficiently defined.

**Methods:**

In this study, we integrated bulk RNA-seq (GSE38417, GSE109178), small RNA-seq (GSE157668), and single-cell RNA-seq (GSE213925) data and combined differential expression analysis, target gene prediction, functional pathway enrichment mapping, and protein-protein interaction network analysis to screen candidate miRNA-mRNA axes, which were subsequently validated in mdx mice, C2C12 myoblasts, and primary skeletal muscle cells.

**Results:**

We identified 100 differentially expressed miRNAs (DEMs) in DMD muscle, with miR-30c-5p being the most downregulated and possessing the highest number of predicted targets. Integrated analysis revealed SOCS3 as a key upregulated hub gene targeted by miR-30c-5p. ScRNA-seq showed elevated SOCS3 expression in myocytes from DMD muscle, where SOCS3^+^ cells exhibited enriched inflammatory pathways and suppressed metabolic processes. In mdx mice, miR-30c-5p expression showed a pronounced reduction (P < 0.001). In contrast, SOCS3 expression increased at both the transcriptional (P < 0.001) and translational (P < 0.01) levels. Functional experiments further showed that overexpression of miR-30c-5p reduced SOCS3 expression, whereas inhibition of miR-30c-5p increased SOCS3 levels in C2C12 cells and primary skeletal muscle cells. Dual-luciferase reporter assay further supported direct binding of miR-30c-5p to the SOCS3 3′UTR. In mdx-derived primary skeletal muscle cells, miR-30c-5p restoration was also accompanied by reduced TNF-α and increased IL-10 levels, and rescue experiments supported that these anti-inflammatory effects were at least partly SOCS3-dependent.

**Conclusion:**

These findings suggest that the miR-30c-5p/SOCS3 axis is associated with inflammation- and metabolism-related alterations in DMD and warrants further investigation.

## 1 Introduction

Duchenne muscular dystrophy (DMD) is a severe, X-linked disease that cannot be cured. The condition stems from the dystrophin deficiency, an essential structural protein derived from the *DMD* gene. Loss of dystrophin disrupts sarcolemmal membrane integrity, leading to progressive muscle fiber degeneration, persistent inflammatory infiltration, and connective tissue fibrosis (Duan et al., 2021; Wilson et al., 2022). The birth prevalence is approximately 19.8/100,000 live male infants (Vincik et al., 2024). This disease usually begins between the ages of 2 and 5. Most patients lose the ability to walk independently around the age of 10–12. The average life expectancy of DMD patients is mostly between 20 and 30 years old, with heart and lung failure being the main cause of death (Duan et al., 2021; Bushby et al., 1999; Birnkrant et al., 2018). Although glucocorticoid therapy (Duan et al., 2021; Birnkrant et al., 2018) can slow disease progression to some extent, it is limited by substantial adverse effects and an inability to reverse the underlying pathological processes. In recent years, novel therapeutic strategies, including exon-skipping approaches and micro-dystrophin gene therapy (Komaki, 2025; Łoboda et al., 2025), have broadened the treatment landscape of DMD. Nevertheless, their clinical benefits remain constrained by limitations in delivery, durability, mutation specificity, and incomplete correction of downstream pathological processes. Therefore, further investigation of molecular mechanisms in DMD remains necessary.

When dystrophin is absent, it undermines the structural stability of the sarcolemma in skeletal muscle. This makes muscle fibers more prone to damage, and further leads to disruptions in calcium homeostasis, along with oxidative stress and metabolic disorders. These pathological changes cause a repetitive cycle of muscle fiber necrosis and regeneration (Mareedu et al., 2021; Pétrilli et al., 2007; Gissel, 2005). Simultaneously, damage-associated molecular patterns (DAMPs) activate the immune response of the body, promoting the progression of chronic inflammation and muscle tissue fibrosis (Starosta and Konieczny, 2021; Lemos et al., 2015; Cardone et al., 2023). Recent research indicates that non-coding RNAs, especially microRNAs (miRNAs), contribute to critical processes associated with DMD pathogenesis (Kielbowski et al., 2024; Ballarino et al., 2016; Erriquez et al., 2013), including inflammatory responses, metabolic pathways, and cellular remodeling. However, the exact way this works has not yet been fully figured out.

MiRNAs serve as post-transcriptional regulators and have gained increasing attention in neuromuscular research. They are typically 18–23 nucleotides long and bind to the complementary sequences within the 3′UTR of their target mRNAs, which in turn either suppresses protein synthesis or promotes mRNA degradation (Lee and Ambros, 2001; Lee et al., 1993; Wightman et al., 1993; Das et al., 2014; Diener et al., 2022; Gou et al., 2023). Accumulating evidence shows that numerous miRNAs display abnormal expression patterns in muscle tissues from patients with DMD, as well as in the C57BL/10ScSn-Dmd^mdx^/J (mdx) mouse model. For example, muscle-specific miRNAs such as miR-133b (Taetzsch et al., 2021) are activated during myofibril injury and participate in regeneration-related signal transduction after injury; while the miR-29 (Bier et al., 2018) family, which has anti-fibrotic effects, is significantly reduced in DMD muscle, leading to excessive collagen deposition and extracellular matrix remodeling. Another highly expressed miR in muscle tissue, miR-206, is thought to promote myoblast differentiation and enhance myotrophic protein expression by regulating Notch3, Akt, and other signaling pathways, thereby promoting regeneration (Liu et al., 2012; Amirouche et al., 2017; Soblechero-Martín et al., 2021; Boppart et al., 2011). In addition, miR-486 (Samani et al., 2022; Yedigaryan and Sampaolesi, 2021), a muscle-enriched miRNA, plays a critical role in muscle repair, myogenesis, satellite cell activation and differentiation, and protein turnover, further supporting the importance of myomiRs in DMD. However, most miRNA studies concentrate on individual miRNAs or specific pathways. Exploration of the broader miRNA–mRNA regulatory network remains limited. At the same time, high-throughput sequencing technologies now offer effective approaches to investigate the biological processes of complex diseases. scRNA-seq studies related to DMD have revealed the heterogeneity of fibrotic/adipogenic precursor cells (FAPs) and their dynamic trajectory of differentiation into fibroblasts (Fernandez-Simon et al., 2024). However, systematic studies combining single-cell transcriptome results with miRNA-mRNA regulatory networks are still rare.

**FIGURE 1:**
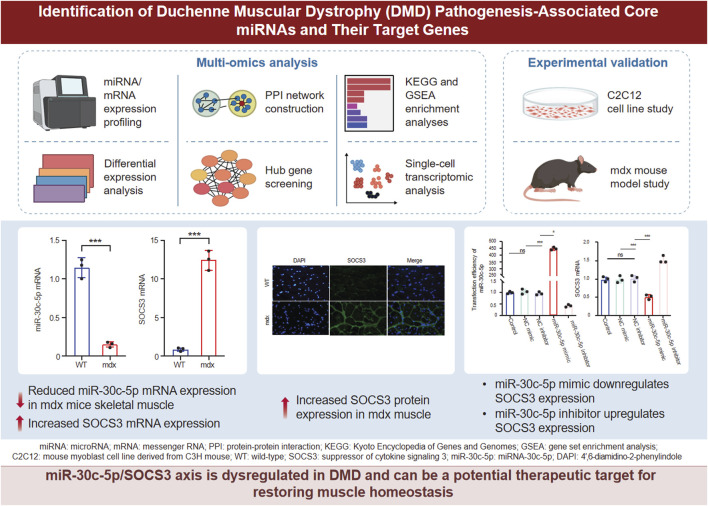
Experimental flow.

Therefore, we combined bioinformatics analysis and experimental verification to identify key miRNAs and mRNAs associated with DMD and analyzed their enrichment pathways. Our aim was to dissect the molecular pathways driving inflammation and fibrosis in DMD, concentrating on the miRNA–mRNA regulatory axis. We conducted experimental analyses using animal tissues, C2C12 myoblasts, and primary skeletal muscle cells for the identification of the key molecular mechanisms that underlie inflammation and metabolic disorders in DMD (Figure 1). The findings can guide the development of miRNA-based therapeutic strategies.

## 2 Methods

### 2.1 Sources of miRNA and mRNA expression profile data

Herein, publicly available DMD-associated multi-omics expression datasets were retrieved from the GEO repository, including miRNA sequencing data, mRNA transcriptome data from human skeletal muscle tissues, and scRNA-seq data from mice. After downloading, the data were processed according to the annotation information from each GEO platform, including format conversion, extraction of the expression matrix, and standardization preprocessing, thereby maintaining comparability and data quality across different platforms.

The miRNA sequencing dataset GSE157668 was generated on the Applied Biosystems SOLiD 5500 platform and included eight biopsy samples of skeletal muscle from DMD patients and three age-matched control subjects. The dataset was analyzed to identify differentially expressed miRNAs (DEMs) in DMD at the initial screening stage. The results subsequently informed the prediction of target genes and the enrichment analysis of relevant biological pathways.

The gene expression microarray datasets GSE38417 and GSE109178 were generated using the Affymetrix Human Genome U133 Plus 2.0 Array platform. Dataset GSE38417 comprises muscle biopsy samples obtained from boys diagnosed with DMD and healthy controls matched by age. Dataset GSE109178 contains transcriptome data from 49 human muscle biopsy samples, representing various neuromuscular disease states, including normal controls (n = 6), DMD (n = 17), Becker muscular dystrophy (n = 11), and several subtypes of limb-girdle muscular dystrophy (n = 15). For this study, only samples from the normal control and DMD groups were selected for differential expression analysis. Combined with the predicted miRNA target gene information, an miRNA-mRNA regulatory network was built.

In GSE213925, scRNA-seq of mouse muscle was performed using the 10X Genomics Chromium platform, including samples from healthy (wt-NSG, n = 2) and severe DMD model (mdxD2-NSG, n = 2) mice.

### 2.2 Differential expression analysis

Differential expression profiling was performed independently on both the miRNA and mRNA datasets, utilizing the limma package in R (v4.4.2) to identify significant changes in expression. To ensure reliable analysis of miRNA expression, features with low expression levels were excluded, retaining only miRNAs that were expressed in at least 80% of all samples. This threshold was used to reduce the influence of sporadically detected or unstable features in a small cohort while preserving miRNAs with relatively robust expression across samples. Library sizes underwent normalization via the TMM method. Expression values were then transformed to log_2_ counts per million [log_2_(CPM + 1)]. DEMs and genes were defined using the criteria of FDR < 0.05 and |log_2_FC| ≥ 1. The resulting DEMs were used for target gene prediction analysis. The two transcriptomic datasets were independently screened for differentially expressed genes (DEGs). Subsequently, the intersected DEGs exhibiting consistent directional changes was identified and retained as a candidate core set for subsequent network and pathway analyses.

### 2.3 miRNA target prediction and integrative analysis

Predicted target genes corresponding to the selected DEMs were collected. The data were sourced from miRTarBase (Huang et al., 2022) and miRDB (Chen and Wang, 2020). In miRTarBase, only experimentally validated miRNA–target interactions were retained. For miRDB, target predictions with a score ≥80 were retained. MiRNAs exceeding 2,000 predicted targets were subsequently removed. The resulting target genes were obtained. They were then matched against the DEGs that were common across both transcriptomic datasets. Through this filtering strategy, we identified high-confidence miRNA–mRNA regulatory pairs that were substantiated by experimentally validated or computationally high-scoring interaction evidence, as well as by concordant expression alterations across multiple datasets.

### 2.4 Functional enrichment analysis

Functional enrichment analysis of the selected target genes was performed using KEGG pathway analysis with the clusterProfiler package in R. Enrichment was considered statistically significant at an FDR < 0.05.

### 2.5 PPI network

To characterize interactions among the candidate genes, a PPI network was generated with the STRING database, restricting the analysis to *Homo sapiens* and using 0.7 as the minimum interaction score. The resulting network was loaded into Cytoscape (v3.10.3) for visualization as well as downstream analyses. Node importance was evaluated with the cytoHubba plugin using three topological methods, namely, Degree, MCC, and EPC. Genes showing consistently high rankings across these approaches were retained as potential hub genes for downstream mechanistic investigation and experimental validation.

### 2.6 Single-cell RNA-seq analysis

#### 2.6.1 Data preprocessing and quality control

We imported the raw data from 10×Genomics using the Read10X function in the Seurat package and constructed a Seurat object with the CreateSeuratObject function. Meanwhile, we performed quality control based on the median absolute deviation (MAD), eliminating cells with abnormal nCount_RNA, nFeature_RNA, and mitochondrial transcript levels. Then, we used the scDblFinder package to remove doublet cells, reducing the impact of technical bias on subsequent analyses.

#### 2.6.2 Normalization, dimensionality reduction, and batch correction

We normalized and scaled the merged expression matrix following the standard Seurat workflow, which includes normalization and standardization steps. We selected the 2000 most variable genes as the input for principal component analysis (PCA). To address batch effects, we used the Harmony software package for correction. Subsequently, we chose the first 40 harmonized dimensions to support subsequent downstream analytical processes.

#### 2.6.3 Clustering and cell type annotation

The Louvain algorithm (resolution = 0.2) was utilized to cluster cells, followed by dimensionality reduction and visualization through UMAP, facilitating the exploration of cellular heterogeneity. Major cell types, including muscle satellite cells (MuSCs), myocytes (MC), endothelial cells (EC), macrophages, pericytes, tenocytes, neutrophils, mast, Schwann cells, and stromal cells, were annotated based on canonical marker genes.

#### 2.6.4 Cell type-specific differential expression and SOCS3^+^ MC subset analysis

Differential gene expression between DMD and control groups was analyzed within each cell type using the FindMarkers function (Wilcoxon test; |log_2_FC| ≥ 1, FDR < 0.05). Upregulated genes were intersected with the final predicted targets of downregulated miRNAs to identify potential regulatory genes.

The MC cluster was further stratified into SOCS3-high (SOCS3^+^) and SOCS3-low (SOCS3^−^) subsets based on the median expression level of SOCS3. Subset-specific DEGs were identified using the same FindMarkers function (|log_2_FC| ≥0.58, P < 0.05) and subjected to pathway enrichment analysis. DEGs were ranked by log_2_FC, and enrichment analysis was conducted using clusterProfiler package with MSigDB Hallmark and KEGG gene sets. Significantly enriched pathways were defined by FDR < 0.05.

### 2.7 *In vivo* animal model and skeletal muscle sample collection

Individual animal experiments were performed with full adherence to national and institutional animal welfare standards, and were carried out with approval of the IACUC (protocol: 2022YFC2703600). The mice were maintained in SPF conditions. The experimental group was 6-week-old C57BL/10ScSn-Dmdmdx/J (mdx) mice (n = 3), and the age-matched WT controls (n = 3) were obtained at Shanghai Model Organisms Center, Inc. (Shanghai, China). Mouse gastrocnemius muscles were collected for molecular and histological analyses. The cross-species conservation of the miR-30c-5p binding site in the SOCS3 3′UTR was confirmed using TargetScan, providing evidence that this murine model accurately reflects human translational biology (Supplementary Material S1).

### 2.8 Primary skeletal muscle cell isolation

Gastrocnemius WT and mdx mice of 6 weeks old were used as primary myogenic cells sources. Immediately after dissection, muscle tissues were finely minced and subjected to enzymatic digestion in 0.2% collagenase II and 0.05% trypsin at 37 °C with gentle shaking. We first passed the prepared cell suspension through a 70-μm strainer, followed by centrifugation at 500 *g* for 5 min. After centrifugation, we collected the cells, transferred them into growth medium, and plated them in standard culture dishes. After incubating for about 2 h, we replaced the medium to remove adherent fibroblasts. Finally, we collected the non-adherent muscle-derived cells and seeded them into new culture dishes.

### 2.9 Cell culture and transfection

The murine C2C12 myoblast cell line was sourced from the Cell Bank of the Chinese Academy of Sciences. For experimental use, cells were propagated in high-glucose DMEM containing 10% fetal bovine serum, 1% penicillin-streptomycin for routine cell culture maintenance. Cells were cultured at 37 °C in a humidified, 5% CO_2_ atmosphere, ensuring optimal growth and viability.

The isolated primary skeletal muscle cells were cultured in complete DMEM containing 10% FBS, 1% penicillin-streptomycin, and basic fibroblast growth factor (bFGF; Gibco). This medium formulation supported optimal cell attachment and proliferation for downstream experiments. We cultured the cells in a humidified incubator set at 37 °C with 5% CO_2_. The DMEM, FBS, and bFGF used in the experiments came from Gibco.

For transfection, C2C12 cells and primary skeletal muscle cells were plated in 6-well plates at 2.0 × 10^5^ cells per well. Transfection was initiated when cell confluence reached 70%–80% using Lipofectamine 3000 (Invitrogen, MA, United States). Following the recommended protocol, miRNA mimics were applied at 50 nM, while miRNA inhibitors were used at 100 nM.

C2C12 cells were divided into five groups: Control (no transfection), miR-30c-5p mimic, NC-mimic, miR-30c-5p inhibitor, and NC-inhibitor. The mimic/inhibitor groups were transfected with corresponding oligonucleotides, and the NC groups with negative controls.

To examine whether the anti-inflammatory effects of miR-30c-5p were mediated by SOCS3, loss- and gain-of-function experiments were performed in primary skeletal muscle cells. The experimental groups were WT, mdx, miR-30c-5p mimic, miR-30c-5p inhibitor, si-SOCS3, miR-30c-5p inhibitor + si-SOCS3, SOCS3 overexpression, and miR-30c-5p mimic + SOCS3 overexpression. WT and mdx cells were isolated from wild-type and mdx mice, respectively, whereas the remaining groups were established by transfecting mdx-derived primary skeletal muscle cells with the corresponding oligonucleotides or expression construct. We replaced the medium 24 h after transfection, and collected the cells 48 h post-transfection for subsequent analysis.

All synthetic oligonucleotides and corresponding negative controls were purchased from MedChemExpress (MCE, United States). The siRNA targeting mouse SOCS3 and a mouse SOCS3 overexpression plasmid were obtained from GenePharma (Shanghai, China). Detailed sequence and construct information are provided in Supplementary Material S2.

### 2.10 Dual-luciferase reporter assay

The SOCS3 3′UTR fragment containing the predicted miR-30c-5p binding site was cloned into the pmirGLO vector to generate the SOCS3-WT-3′UTR reporter. A mutant reporter with substitutions in the seed-binding region was generated in parallel and named SOCS3-MUT-3′UTR. HEK293T cells were co-transfected with the reporter plasmids and either miR-30c-5p mimic or NC mimic using Lipofectamine 3000 (Invitrogen, MA, United States). After 24 h, firefly and Renilla luciferase activities were measured using a dual-luciferase reporter assay kit (Beyotime, China; Cat# RG028).

### 2.11 qRT-PCR

Total RNA was obtained from gastrocnemius muscle tissue. RNA from collected C2C12 cells was similarly isolated using TRIzol® Reagent (Ambion, United States; Cat# 15596018). miRNA samples were reverse-transcribed to cDNA using the stem-loop–based miRNA 1st Strand cDNA Synthesis Kit (Vazyme, Nanjing, China; Cat# MR101). In contrast, mRNA was transcribed into cDNA employing HiScript® III All-in-one RT SuperMix (Vazyme, Nanjing, China; Cat# R333-01), enabling subsequent quantitative analyses. Quantitative PCR was performed with 2× SYBR Green qPCR Mastermix (YangGuangBio, Beijing, China; Cat# C210101). Primer sequences are provided in Supplementary Material S2, and all primers were synthesized by Beijing Liuhe BGI Genomics Co., Ltd. (Beijing, China). The expression of SOCS3 mRNA was calibrated against β-actin, whereas miR-30c-5p levels were calibrated against U6 to ensure accurate quantification in qRT-PCR analyses. Relative expression was calculated using the 2^−ΔΔCt^ method.

### 2.12 WB

Protein concentration was measured using the BCA Protein Assay Kit (YangGuangBio, Beijing, China). Proteins (30 μg per sample) were first separated by SDS-PAGE (ACE Biotechnology, Nanjing, China) and subsequently immobilized onto PVDF membranes to enable immunoblotting analyses. The membranes were first blocked. They were subsequently incubated overnight at 4 °C with primary antibodies against SOCS3 (1:1,000, Proteintech, Wuhan, China; Cat# 66797-1-Ig), β-actin (1:1,000, Affinity Biosciences, China; Cat# AF7018) and GAPDH (1:1,000, YangGuangBio, China; Cat# PM0001). The membranes underwent three washes with PBST. They were then incubated with HRP-conjugated secondary antibody (YangGuangBio, Beijing, China; Cat# C081801). Protein bands were detected with an ECL substrate and captured using a ChemiScope 6100 imaging system (CLINX, Shanghai, China). Band intensities were measured using ImageJ software.

### 2.13 ELISA

TNF-α and IL-10 were measured by ELISA in culture supernatants from WT primary skeletal muscle cells, mdx primary skeletal muscle cells, and the indicated intervention groups established from mdx-derived primary skeletal muscle cells. WT cells served as the baseline control and were not subjected to matched transfection conditions. The concentrations of TNF-α and IL-10 were then measured using commercial ELISA kits (Chengzhikewei, China), strictly following the manufacturer’s recommended procedures. Cytokine concentrations were calculated using standard curves.

### 2.14 Immunofluorescence analysis

Frozen muscle sections were treated with 4% PFA for fixation, rinsed with PBS, and permeabilized using 0.1% Triton X-100. Primary antibody against SOCS3 (Proteintech, Wuhan, China; Cat# 66797-1-Ig) was applied overnight at 4 °C. Following three 5-min washes in PBST, sections were incubated for 1 h at ambient temperature in dark with a fluorophore-labeled secondary antibody (YangGuangBio, Beijing, China; Cat# C081805; dilution 1:500). Nuclei were counterstained with DAPI (Cat# C190303, 1 μg/mL, YangGuangBio, Beijing, China) for 15 min. Fluorescence images were acquired under identical microscope settings for all groups to allow comparison of SOCS3 localization and signal intensity.

All the experiments were conducted in three independent biological replicates. Each replicate consisted of n = 3 samples.

### 2.15 Statistical analysis

R (v4.4.2) was used for bioinformatic analyses. Differential miRNA and mRNA expression was analyzed using the limma package, with |log_2_FC| ≥1 and FDR < 0.05 as the cutoff criteria. Single-cell RNA-seq data were processed using Seurat and Harmony, and differences between cell subsets were assessed using the Wilcoxon rank-sum test. Functional enrichment analysis was carried out with clusterProfiler.

Experimental results were processed in GraphPad Prism (v10.4.1). Parametric comparisons across multiple groups were performed via one-way ANOVA with Dunnett’s *post hoc* correction for normally distributed data, whereas non-normally distributed variables were analyzed using the nonparametric Kruskal–Wallis test. Continuous outcomes are presented as mean ± standard deviation, and statistical significance was defined as P < 0.05 (two-tailed). Densitometric quantification of WB bands was carried out in ImageJ, and graphical representation of results was generated using both GraphPad Prism and R software.

## 3 Results

### 3.1 Screening of DEGs and functional enrichment analysis

**FIGURE 2:**
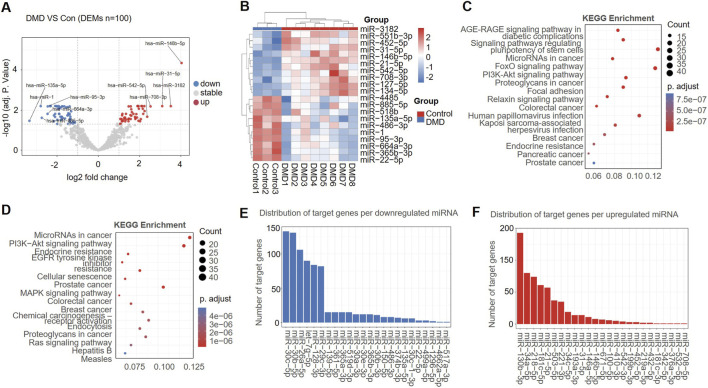
Differentially expressed miRNAs (DEMs) and functional interpretation in Duchenne muscular dystrophy (DMD). **(A)** Volcano plot. **(B)** Heatmap of DEMs. **(C)** KEGG pathway enrichment for targets of downregulated miRNAs. **(D)** KEGG pathway enrichment for targets of upregulated miRNAs. **(E)** Target load per downregulated miRNA. **(F)** Target load per upregulated miRNA.

After removing non-miRNA sequences and low-expression entities, a total of 726 reliably expressed miRNAs were retained for differential expression analysis. This analysis identified 100 significantly DEMs, comprising 49 upregulated and 51 downregulated miRNAs (Figure 2A). A volcano plot illustrated the distribution of all DEMs (Figure 2A), and a heatmap further delineated the expression patterns of the top 20 DEMs between the DMD and the healthy control group (Figure 2B).

We used the miRTarBase (Huang et al., 2022) and miRDB (Chen and Wang, 2020) databases to identify key regulatory molecules, target gene prediction for the DEMs, and only overlapping targets supported by both resources were retained for subsequent analysis. We analyzed the KEGG pathways enriched by the targets of downregulated miRNAs, and found that these targets are significantly involved in multiple signaling pathways. These include the AGE-RAGE pathway (linked to diabetic complications), FoxO signaling, PI3K–Akt signaling, focal adhesion, human papillomavirus infection, and TNF signaling (Figure 2C). These enriched pathways are closely associated with chronic inflammation, tissue remodeling, aberrant immune responses, and fibrotic processes, indicating that the downregulated miRNAs likely exert critical modulatory influence over key disease-driving mechanisms in DMD.

KEGG assessment of 778 target genes for upregulated miRNAs was performed. The results showed that these target genes were enriched in the EGFR tyrosine kinase inhibitor resistance, as well as the PI3K–Akt, MAPK and Ras signaling pathways (FDR<0.05) (Figure 2D). These pathways are broadly involved in cell proliferation, survival, and signal transduction regulation.

Analysis of predicted target genes across downregulated miRNAs revealed that miR-30c-5p has the largest number of targets (Figure 2E), emphasizing its role as a potentially pivotal miRNA with widespread regulatory effects in the network. Figure 2F displays the distribution of predicted target genes for all upregulated miRNAs.

In summary, the target genes of downregulated miRNAs participate prominently in DMD-related pathological pathways, such as inflammation, immune regulation, and extracellular matrix remodeling. Among these candidates, miR-30c-5p was prioritized for further study because it showed marked downregulation, had the largest number of predicted target genes, and was linked to pathways closely related to DMD pathophysiology.

### 3.2 Integrated analysis of DEMs target genes and transcriptomic data

**FIGURE 3:**
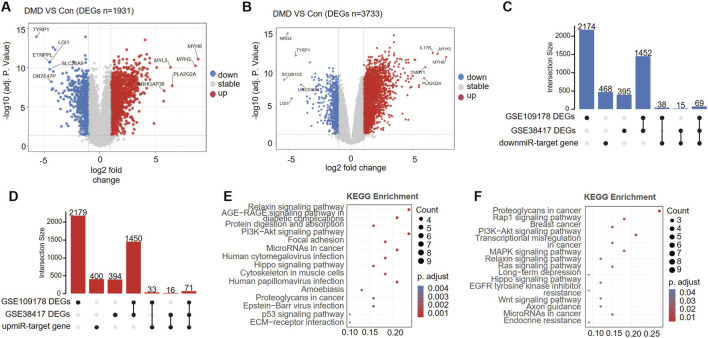
Differentially expressed genes (DEGs) and functional interpretation in Duchenne muscular dystrophy (DMD). **(A,B)** Volcano plots for two cohorts showing differential expression between DMD and controls in GSE38417 **(A)** and GSE109178 **(B)**. **(C,D)** Overlap between DEGs and miRNA targets (UpSet plots). Intersections among DEGs from GSE109178, DEGs from GSE38417, and predicted targets of downregulated miRNAs **(C)** or upregulated miRNAs **(D)**. **(E,F)** KEGG pathway enrichment of the overlapping gene sets. Enrichment analysis for genes in **(C)** (**E**, down-miRNA targets ∩ DEGs) and in **(D)** (**F**, up-miRNA targets ∩ DEGs).

To further clarify the functional interactions between DEMs and their potential mRNA targets in DMD muscle tissue, we analyzed two independent public transcriptomic datasets, GSE38417 and GSE109178. Differential expression analysis revealed 1,931 significant DEGs in GSE38417, including 1,272 upregulated and 659 downregulated genes (Figure 3A), and 3,733 DEGs in GSE109178, comprising 3,089 upregulated and 644 downregulated genes (Figure 3B).

We further conducted an intersection operation between the predicted target gene set of DEMs and DEGs from two transcriptome datasets. Visualization using UpSet plots indicated that 69 predicted targets of downregulated miRNAs exhibited altered expression in both datasets (Figure 3C). In contrast, the intersection of target genes for upregulated miRNAs included 71 genes (Figure 3D), yielding a high-confidence candidate gene set suitable for subsequent functional enrichment analyses.

KEGG analysis of the overlapping targets associated with the downregulated miRNAs highlighted several pathways related to inflammation, immune activity, and extracellular matrix remodeling. The enrichment analysis highlighted several key pathways, notably relaxin signaling, the AGE–RAGE signaling pathway implicated in diabetic complications, PI3K–Akt signaling, protein digestion and absorption, focal adhesion, and ECM–receptor interactions (Figure 3E). These findings indicate that downregulated miRNAs may participate in the inflammatory, immune, matrix-remodeling, and metabolic disturbances observed in DMD skeletal muscle through coordinated effects on multiple pathways.

The overlapping targets for upregulated miRNAs were mainly mapped to the PI3K–Akt, MAPK, Ras, and relaxin signaling pathways. They were also associated with EGFR tyrosine kinase inhibitor resistance (Figure 3F). Collectively, these signaling pathways contribute to the modulation of cellular processes, including proliferation, survival, adaptation to stress, and the regulation of intracellular signaling networks.

### 3.3 PPI network analysis

**FIGURE 4:**
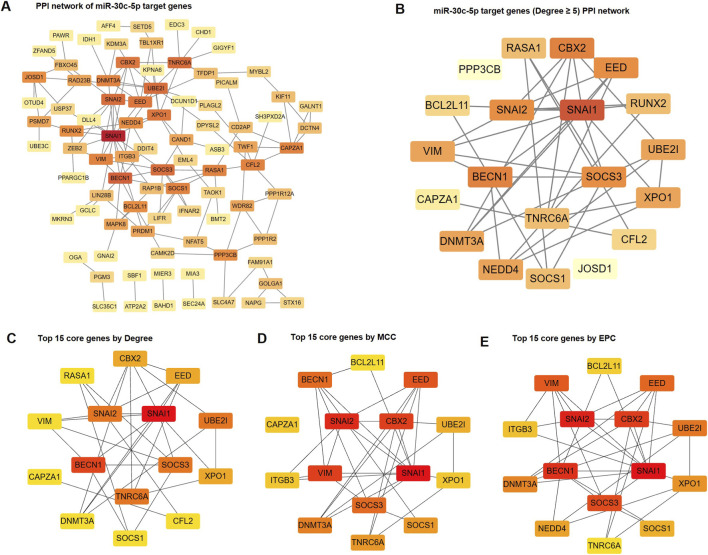
Protein–protein interaction (PPI) network analysis and screening of core genes. **(A)** PPI network of all target genes of downregulated miR-30c-5p. **(B)** PPI network of target genes with a Degree value of ≥5. **(C–E)** The top 15 core genes identified using Degree **(C)**, Maximal Clique Centrality (MCC) **(D)**, Edge Percolated Component (EPC) **(E)** algorithms using the CytoHubba plugin in Cytoscape.

To identify key regulatory targets among the downregulated miRNAs in DMD, we focused on miR-30c-5p and analyzed its computationally predicted downstream target genes. We submitted the set of predicted target genes to the STRING database (https://cn.string-db.org/) (Szklarczyk et al., 2021) for PPI network generation. The initial PPI network encompassed 87 nodes and 137 edges (Figure 4A, Supplementary Material S3). We then used a Degree threshold of ≥5 as the filtering standard to extract a relatively core subnetwork, which consisted of 20 nodes and 37 edges (Figure 4B).

We further analyzed the PPI network using the CytoHubba plugin integrated in Cytoscape software to identify hub regulators potentially involved in key pathological processes of DMD (Shannon et al., 2003). We adopted the classic analytical algorithms, such as Degree, MCC, and EPC (Chin et al., 2014) to rank and screen for hub genes. A final set of the top 15 hub genes was obtained (Figures 4C–E, Supplementary Materials S4, S5), including SOCS3, BECN1, SNAI1, and VIM, which consistently appeared across multiple algorithms, suggesting high network centrality and biological significance.

### 3.4 Construction of miRNA–mRNA regulatory network and identification of key miRNAs

**FIGURE 5:**
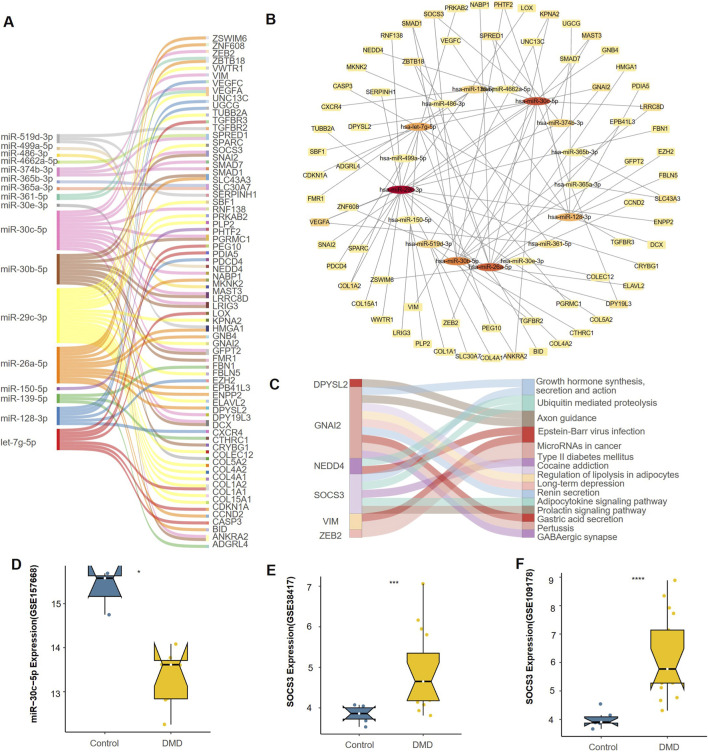
miRNA–mRNA regulatory network analysis and identification of hub miRNAs. **(A)** Contribution of each downregulated miRNA. **(B)** miRNA–mRNA regulatory network of downregulated miRNAs and core genes identified from the PPI network. **(C)** KEGG pathway enrichment of the final set of miR-30c-5p target genes. **(D)** Expression of miR-30c-5p in skeletal muscle (GSE157668). **(E,F)** Expression of SOCS3 in GSE38417 and GSE109178, respectively. *P < 0.05, **P < 0.01, ***P < 0.001.

Based on differential expression analysis and target gene prediction results, we integrated the final differentially expressed downregulated miRNAs, target gene prediction results, and PPI network to construct a miRNA-mRNA regulatory network (Figures 5A,B). A Sankey diagram shows each downregulated miRNA and its corresponding target gene (Figure 5A), while upregulated miRNAs and their predicted targets were also analyzed in parallel (Supplementary Material S6). Among these, miR-30c-5p exhibited the highest number of computationally predicted target genes. In addition to miR-30c-5p, other significantly downregulated miRNAs—most notably miR-30b-5p and miR-26a-5p—were prominently enriched in the integrated miRNA–mRNA regulatory network, indicating their potential involvement in DMD-associated molecular pathways.

Furthermore, a PPI network was established between these key miRNAs and their shared target genes (Figure 5B). The analysis indicated that miR-30c-5p targets multiple network hub genes, including SOCS3, DPYSL2, NEDD4, VIM, and ZEB2, indicating it may play an important regulatory role in DMD.

Functional KEGG analysis of the core miR-30c-5p target genes (Figure 5C) demonstrated predominant involvement in pathways governing neuromuscular development, metabolic processes, and intracellular signaling. Key pathways included axon guidance, regulation of adipocyte lipolysis, growth hormone synthesis, secretion, and action, type II diabetes mellitus, and microRNAs implicated in cancer. These findings further support the multi-target regulatory potential of miR-30c-5p in DMD pathogenesis.

Expression analysis using the GSE157668 dataset demonstrated that miR-30c-5p was substantially reduced in skeletal muscle from DMD patients (FDR < 0.05, Figure 5D). Concurrently, its principal target, SOCS3, exhibited pronounced upregulation in both GSE38417 and GSE109178 datasets (FDR<0.001, Figures 5E,F), underscoring a potential inverse regulatory relationship. The expression of miR-30c-5p and SOCS3 displayed an opposing pattern. This observation reflects the canonical negative regulation by miRNAs and further supports the functional significance of the miR-30c-5p/SOCS3 axis. Our findings are supported by earlier evidence showing that miR-30c-5p directly interacts with SOCS3. This interaction participates in the modulation of both inflammatory and metabolic signaling in several disease contexts (Bin et al., 2024; Hu et al., 2023).

The extensive target range of miR-30c-5p, its centrality in the PPI network, the functional involvement of its targets in DMD-associated pathways, and its inverse relationship with SOCS3 together underscore the biological significance of the miR-30c-5p/SOCS3 axis and its potential as a therapeutic avenue in DMD.

### 3.5 Single-cell transcriptomic analysis

#### 3.5.1 Single-cell transcriptomic profiling reveals altered cellular composition in DMD muscle

**FIGURE 6:**
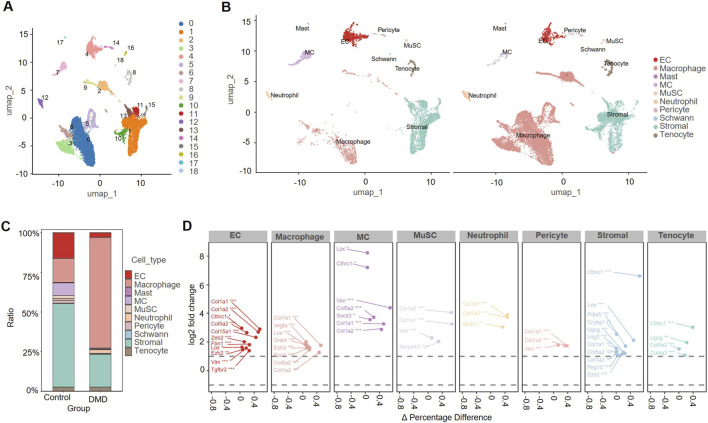
Single-cell transcriptomic profiling of control and Duchenne muscular dystrophy (DMD) skeletal muscle. **(A)** Uniform manifold approximation and projection (UMAP) visualization of unsupervised clustering based on integrated single-cell RNA sequencing data from gastrocnemius muscle, revealing 19 transcriptionally distinct cell populations across control and dystrophic (DMD) samples. **(B)** Annotation of major cell types using canonical marker genes, including endothelial cells (EC), macrophages, mast cells, myocytes (MC), muscle satellite cells (MuSCs), neutrophils, pericytes, Schwann cells, stromal cells (including fibro-adipogenic progenitors and fibroblasts), and tenocytes. **(C)** Stacked bar plot comparing the relative proportions of each cell population between control and DMD groups. **(D)** Dot plots showing cell type-specific upregulation of genes in DMD muscle that overlap with the predicted targets of downregulated miRNAs. Notably, SOCS3 was strongly upregulated in MC. *P < 0.05, **P < 0.01, ***P < 0.001.

To investigate cellular heterogeneity and compositional changes, we integrated scRNA-seq data from four samples in the GSE213925 dataset. After standard quality control and dimensionality reduction, 19 cell clusters (0–18) were identified (Figure 6A). Using classic marker genes, we categorized these cell clusters to 10 major cell types: MuSCs, MCs, macrophages, neutrophils, ECs, Schwann cells, mast cells, stromal cells, tenocytes, and pericytes (Figure 6B). The relative proportions of these cell types were visualized for control and DMD muscle samples (Figure 6C).

To identify potential regulatory relationships, upregulated genes from the single-cell analysis were cross-referenced with the predicted targets of downregulated miRNAs (Figure 6D). Integrated analysis of the overlapping genes was performed. Many of these genes showed enrichment in pathways related to ECM remodeling, inflammatory activation, and cellular stress responses. Notably, SOCS3 was markedly upregulated in DMD myocytes (|log_2_FC| = 3.56, FDR < 0.05) and co-expressed with ECM/stress-associated genes such as Vim and Col1a2.

#### 3.5.2 Differential gene expression between SOCS3^+^ and SOCS3^−^ myocytes subsets

**FIGURE 7:**
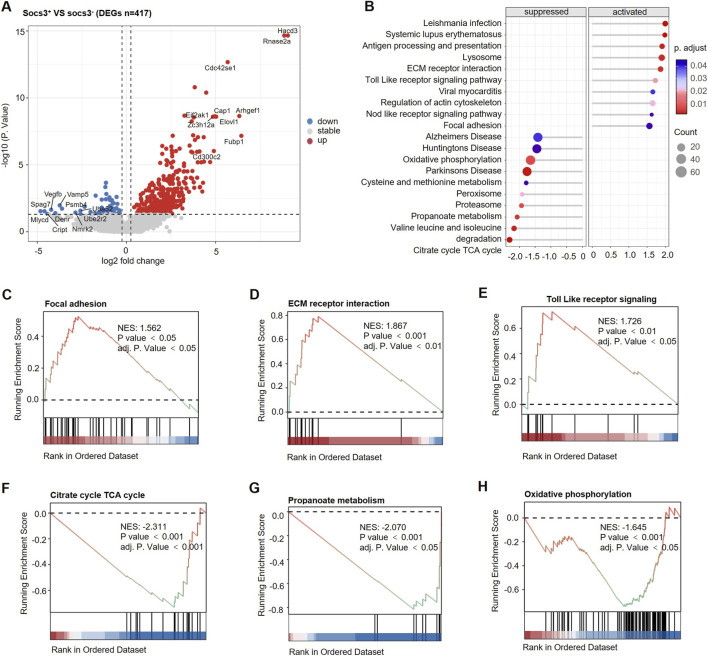
SOCS3^+^ MCs exhibit pro-inflammatory activation and metabolic repression in Duchenne muscular dystrophy (DMD) skeletal muscle. **(A)** Volcano plot of DEGs between SOCS3^+^ and SOCS3^-^ myocyte subsets, revealing 417 significant DEGs (|log_2_FC| ≥ 0.58, FDR < 0.05). **(B)** Gene set enrichment analysis (GSEA) of DEGs showing the top 20 significantly enriched pathways. **(C–E)** GSEA plots showing representative activated pathways in SOCS3^+^ cells: focal adhesion, extracellular matrix (ECM)-receptor interaction, and Toll-like receptor signaling pathway. **(F–H)** GSEA plots showing representative suppressed pathways in SOCS3^+^ cells: tricarboxylic acid (TCA) cycle, propanoate metabolism, and oxidative phosphorylation.

To further characterize the functional role of SOCS3 in MCs, we stratified the MC cluster into SOCS3^+^ and SOCS3^−^ subsets based on expression levels and performed differential gene expression analysis. In SOCS3^+^ MCs, gene expression profiling identified a total of 363 genes showing upregulation and 54 genes showing downregulation (|log_2_FC| >0.58, FDR < 0.05; Figure 7A). Notably upregulated genes included Zc3h12a, Cap1, Elovl1, Hacd3, Cdc42se1, and Rnase2a, many of which are involved in lipid metabolism, stress response, and immune regulation. These findings suggest that SOCS3 upregulation may be linked to the broader activation of metabolic and inflammatory pathways.

#### 3.5.3 Functional enrichment of the SOCS3^+^ myocytes subset

We performed gene set enrichment analysis (GSEA), which showed that the SOCS3^+^ MC subset was significantly enriched in multiple inflammation- and immune-related pathways (Figure 7B), such as focal adhesion (Figure 7C), ECM–receptor interaction (Figure 7D), and Toll-like receptor signaling (Figure 7E). These findings indicate that within the inflammatory microenvironment of DMD, SOCS3^+^ MC may develop immune-like characteristics.

In contrast, several key metabolic pathways were markedly downregulated in SOCS3^+^ MC, including the citrate cycle TCA cycle (Figure 7F), propanoate metabolism (Figure 7G), and oxidative phosphorylation (Figure 7H). These results suggest that SOCS3 upregulation is associated with disrupted energy metabolism and proteostasis, potentially contributing to the pathological remodeling of MC in DMD.

These findings suggest that inflammatory activation, metabolic disorders and matrix stress remodeling are the prominent features of SOCS3^+^ myocytes. Therefore, we propose that SOCS3 may be a potential upstream regulatory factor for the pathological reprogramming of muscle tissue in DMD.

### 3.6 Validation of the miR-30c-5p/SOCS3 axis in DMD models

#### 3.6.1 miR-30c-5p is downregulated while SOCS3 is upregulated in the skeletal muscle of DMD mice

**FIGURE 8:**
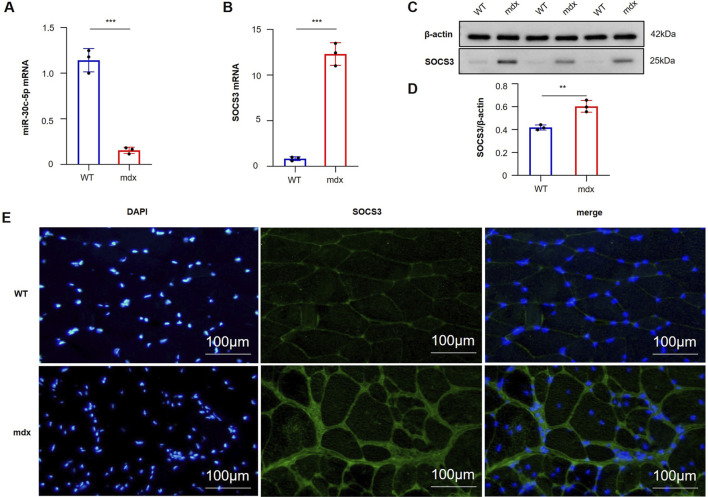
Expression of miR-30c-5p and SOCS3 in skeletal muscle tissues from wild-type (WT) and mdx mice. **(A)** Quantitative reverse transcription polymerase chain reaction (RT-PCR) analysis showing significantly decreased miR-30c-5p expression in the gastrocnemius muscles of mdx mice compared with WT mice. **(B)** SOCS3 mRNA levels were significantly upregulated in mdx mice relative to WT controls. **(C)** Representative Western blot images showing increased SOCS3 protein expression in mdx mouse muscle tissues; β-actin was used as the loading control. **(D)** Densitometric quantification of SOCS3 protein expression normalized to β-actin, showing a significant increase in mdx mice. **(E)** Representative immunofluorescence staining of SOCS3 in skeletal muscle sections. DAPI (blue) stains nuclei; SOCS3 (green) shows cytoplasmic localization. Compared with WT muscles, mdx muscles exhibited markedly increased SOCS3 expression and altered localization patterns (n = 3). **P < 0.01, ***P < 0.001.

In order to confirm the *in vivo* expression differences of miR-30c-5p and SOCS3, quantitative analyses were conducted on gastrocnemius muscle tissues collected from WT and mdx mice. Compared with WT mice, miR-30c-5p expression levels were notably decreased in mdx skeletal muscle (P < 0.001, Figure 8A), whereas SOCS3 showed substantial upregulation at both transcript and protein levels (P < 0.001, Figures 8B–E), in line with the proposed inhibitory effect of miR-30c-5p on SOCS3 expression.

#### 3.6.2 Dual-luciferase reporter assay supports direct binding between miR-30c-5p and SOCS3 3′UTR

**FIGURE 9:**
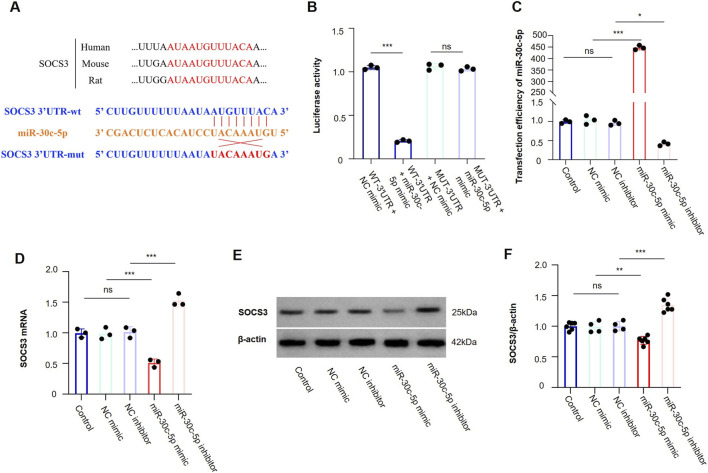
miR-30c-5p directly targets SOCS3 and suppresses its expression. **(A)** Predicted binding site of miR-30c-5p in the SOCS3 3′UTR and sequence conservation across human, mouse, and rat. The wild-type (WT) and mutant (MUT) binding sequences used for luciferase reporter construction are shown. **(B)** The direct interaction between miR-30c-5p and SOCS3 was verified by dual-luciferase reporter assay. **(C)** qRT-PCR analysis confirmed the transfection efficiency of miR-30c-5p in C2C12 cells. **(D)** Relative expression of SOCS3 mRNA in the same groups. **(E)** Western blot showing SOCS3 protein levels. **(F)** Quantification of SOCS3 protein expression normalized to β-actin. ns, not significant; *P < 0.05, **P < 0.01, ***P < 0.001.

Dual-luciferase assay further supported direct binding between miR-30c-5p and SOCS3 (Figures 9A,B). Compared with the SOCS3-WT-3′UTR + NC mimic group, relative luciferase activity was significantly reduced in the SOCS3-WT-3′UTR + miR-30c-5p mimic group, whereas this effect was markedly attenuated in the SOCS3-MUT-3′UTR reporter group. These findings support direct interaction between miR-30c-5p and the predicted binding site in the SOCS3 3′UTR.

#### 3.6.3 miR-30c-5p regulates SOCS3 expression in C2C12 myoblasts

To validate the regulatory interaction of miR-30c-5p with SOCS3, C2C12 myoblasts were transfected with a synthetic miR-30c-5p mimic, a sequence-specific miR-30c-5p inhibitor, or corresponding scrambled control oligonucleotides. Transfection with the mimic led to a substantial upregulation of miR-30c-5p levels, whereas the inhibitor produced the opposite effect (Figure 9C). Functionally, overexpressing miR-30c-5p notably decreased SOCS3 levels, whereas suppressing miR-30c-5p contributed to a substantial increase in SOCS3 levels (Figures 9D–F).

#### 3.6.4 SOCS3-dependent rescue experiments support the anti-inflammatory effect of miR-30c-5p in mdx-derived primary skeletal muscle cells

**FIGURE 10:**
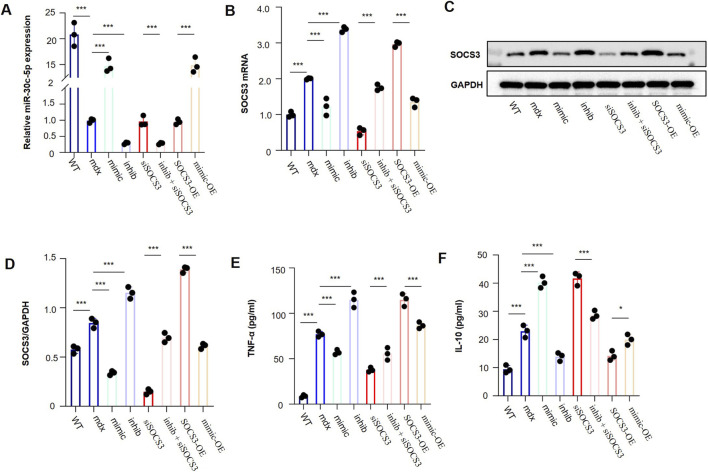
SOCS3-dependent rescue experiments support the anti-inflammatory effect of miR-30c-5p in mdx-derived primary skeletal muscle cells. **(A)** Relative miR-30c-5p expression measured by qRT-PCR in WT primary skeletal muscle cells, mdx primary skeletal muscle cells, and the indicated intervention groups. **(B)** Relative SOCS3 mRNA expression measured by qRT-PCR in the same groups. **(C)** Representative Western blot showing SOCS3 protein expression, with GAPDH used as the loading control. **(D)** Densitometric quantification of SOCS3 protein expression normalized to GAPDH. **(E)** TNF-α levels in culture supernatants measured by ELISA. **(F)** IL-10 levels in culture supernatants measured by ELISA. The intervention groups were established from mdx-derived primary skeletal muscle cells and included miR-30c-5p mimic, miR-30c-5p inhibitor, si-SOCS3, miR-30c-5p inhibitor + si-SOCS3, SOCS3 overexpression (SOCS3-OE), and miR-30c-5p mimic + SOCS3 overexpression. ns, not significant; *P < 0.05, **P < 0.01, ***P < 0.001.

Compared with WT primary skeletal muscle cells, mdx-derived primary skeletal muscle cells showed lower miR-30c-5p expression (Figure 10A), higher SOCS3 expression (Figures 10B–D), and increased TNF-α and IL-10 levels (Figures 10E,F). miR-30c-5p mimic increased miR-30c-5p expression, whereas miR-30c-5p inhibitor further reduced it. Correspondingly, miR-30c-5p overexpression reduced SOCS3 expression, decreased TNF-α, and further increased IL-10, whereas miR-30c-5p inhibition showed the opposite pattern. Silencing SOCS3 produced similar changes, while SOCS3 overexpression showed the reverse trend. In addition, SOCS3 knockdown partially reversed the pro-inflammatory changes induced by miR-30c-5p inhibition, whereas SOCS3 overexpression attenuated the anti-inflammatory effect of miR-30c-5p overexpression. These findings suggest that the anti-inflammatory effect of miR-30c-5p is at least partly mediated through SOCS3.

## 4 Discussion

DMD remains a complex disease characterized by persistent inflammation, metabolic disturbance, and impaired muscle regeneration, underscoring the need to better define the molecular pathways involved in its progression. Through the integration of miRNA, bulk transcriptomic, and scRNA-seq data, we identified the miR-30c-5p/SOCS3 axis as a candidate regulatory pathway associated with these alterations in DMD. miR-30c-5p was markedly reduced in the skeletal muscle of mdx mice, consistent with previous findings in muscle samples from patients with DMD (Trifunov et al., 2020). SOCS3, which has previously been reported to be regulated by miR-30c-5p (Bin et al., 2024; Hu et al., 2023), was consistently upregulated across transcriptomic datasets, mdx muscle tissues, and cell-based models.

Our dual-luciferase reporter analysis further supported direct binding of miR-30c-5p to the SOCS3 3′UTR, and gain- and loss-of-function experiments in C2C12 myoblasts confirmed that miR-30c-5p negatively regulates SOCS3 expression. More importantly, rescue experiments in primary skeletal muscle cells isolated from mdx mice showed that the inflammatory changes associated with miR-30c-5p modulation were at least partly dependent on SOCS3. Notably, miR-30b-5p and miR-26a-5p were also prominently represented in the integrated miRNA-mRNA regulatory network, suggesting that they may also be relevant in DMD. Their possible roles in inflammation, extracellular matrix remodeling, and metabolic adaptation deserve further study.

miR-30c-5p, a member of the miR-30 family, regulates a range of physiological and pathological processes, encompassing fibrosis (Duisters et al., 2009), myocardial ischemia (Wang et al., 2020), inflammation (Ceolotto et al., 2017), and apoptosis (Li et al., 2010). It can act together with miR-133 to inhibit connective tissue growth factor (CTGF) expression, thereby regulating collagen production and ECM remodeling (Duisters et al., 2009). In acute kidney injury, miR-30c-5p was shown to ameliorate apoptosis by suppressing SOCS3 and stabilizing hypoxia-inducible factor 1-alpha (HIF1α) expression (Zou et al., 2017). Moreover, miR-30c-5p modulates angiogenic processes via the SOCS3–STAT3–VEGFA axis (Hu et al., 2023), reinforcing its multifaceted involvement in coordinating inflammatory responses, metabolic homeostasis, and fibrotic tissue restructuring.

SOCS3 was upregulated in both transcriptome datasets and was identified as a core node in the miR-30c-5p predicted regulatory network. Through multiple topological algorithms such as degree, MCC, and EPC, the protein interaction network analysis further confirmed the central position of SOCS3 as a key hub gene. SOCS3 can inhibit cytokine-mediated signal transduction through the JAK/STAT pathway; however, its persistent overexpression in pathological conditions may be closely related to tissue repair defects and exacerbated inflammatory responses in chronic diseases. In metabolic disease models, SOCS3 has been shown to interfere with insulin and leptin signaling, leading to abnormal lipid accumulation, mitochondrial dysfunction, and decreased metabolic flexibility (Steinberg et al., 2006; Yang et al., 2012). These findings provide a theoretical basis for understanding how persistent SOCS3 activation exacerbates muscle pathological damage in DMD.

KEGG analysis of the predicted targets of the downregulated miRNAs revealed that they were mainly enriched in the extracellular matrix receptor interaction and focal adhesion pathways. Single-cell transcriptome data also showed that SOCS3 expression was increased in MC cluster. These cells were divided into SOCS3^+^ and SOCS3^-^ subsets and GSEA enrichment analysis was performed. SOCS3^+^ population was characterized by enrichment of pathways associated with ECM remodeling, inflammatory signaling, and Toll-like receptor (TLR) signaling. Our findings also indicate that SOCS3 upregulation is not just a passive transcriptional alteration, but rather may be associated with an activated inflammatory state in dystrophic muscle cells. In primary skeletal muscle cells derived from mdx mice, restoring miR-30c-5p expression led to decreased TNF-α levels and increased IL-10 levels; conversely, inhibiting miR-30c-5p resulted in the opposite changes. Since TNF-α (Tripodi et al., 2021) is a typical pro-inflammatory cytokine and IL-10 (Nitahara-Kasahara et al., 2021) is commonly recognized as an anti-inflammatory mediator, these changes are consistent with a partial mitigation of the inflammatory phenotype following miR-30c-5p restoration. The rescue experiments strengthen the view that SOCS3 is not merely associated with, but functionally involved in, the inflammatory phenotype observed in dystrophic muscle cells. Together with the enrichment of TLR-related pathways in SOCS3^+^ cells, these findings suggest that SOCS3 may participate in and amplify innate immune activation downstream of dystrophin deficiency and DAMP release in dystrophic muscle (Chen and Nunez, 2010). These findings align with earlier studies showing that TLR4 deficiency markedly attenuates muscle inflammation and functional decline in mdx mice (Bhattarai et al., 2022; Giordano et al., 2015).

In addition to its pro-inflammatory role, SOCS3 is strongly associated with impaired metabolic regulation. Sustained SOCS3 upregulation in skeletal muscle can suppress AMPK and IRS-1/PI3K/Akt pathways, thereby impairing fatty acid β-oxidation, weakening mitochondrial function, and inducing insulin resistance (Steinberg et al., 2006; Yang et al., 2012; Jorgensen et al., 2013; Khattri et al., 2024). Consistent with these reports, SOCS3^+^ myocytes displayed suppression of central metabolic pathways—including oxidative phosphorylation, the TCA cycle, peroxisomal metabolism, and propanoate metabolism. Metabolic impairment is a hallmark of dystrophic muscle (Khattri et al., 2024), and accumulating evidence suggests that metabolic stress may contribute to disease progression by altering the immune–metabolic microenvironment (Milad et al., 2017; Li et al., 2015; Wang et al., 2025).

Prior research indicates that mitochondrial dysfunction occurs at the early stages of DMD skeletal muscle pathology. This event often takes place before noticeable inflammation develops. Hallmarks include abnormal mitochondrial ultrastructure, reduced activity of respiratory chain complexes, diminished membrane potential, and elevated levels of ROS (Moore et al., 2020; Hughes et al., 2019; Spassov et al., 2011). These alterations suggest a pathological basis for subsequent inflammatory and regenerative defects. According to the target enrichment analysis of miR-30c-5p, SOCS3 was linked to multiple energy-related pathways, including regulation of lipolysis, adipocytokine signaling, type II diabetes mellitus, and ubiquitin-mediated proteolysis, the latter being closely connected to mitophagy. These results emphasize SOCS3 as a key regulator of muscle energy homeostasis.

In DMD, the calcineurin–NFAT signaling axis is considered a protective pathway. Previous studies have reported that persistent SOCS3 upregulation may be associated with altered calcineurin-related signaling and impaired mitochondrial adaptation in skeletal muscle (Lebrun et al., 2009; Jorgensen et al., 2013). However, as the present study did not directly assess calcineurin–NFAT activity, mitochondrial function, or downstream adaptive responses, the relevance of these observations to the miR-30c-5p/SOCS3 axis in DMD remains speculative and requires further investigation.

Our cellular experiments further confirmed the regulatory model between miR-30c-5p and SOCS3. C2C12 myoblasts and primary skeletal muscle cells isolated from mdx mice were used to evaluate miR-30c-5p effects. Overexpression of miR-30c-5p substantially decreased SOCS3 transcripts and protein levels. Suppression of miR-30c-5p contributed to higher SOCS3 expression levels.

Although miRNA-based strategies have attracted interest in translational research, their application in DMD remains limited by challenges related to delivery, tissue specificity, and off-target effects (Seyhan, 2024; Diener et al., 2022; Chen et al., 2015). Our findings primarily highlight downstream pathological processes associated with the miR-30c-5p/SOCS3 axis—particularly inflammation, metabolic dysregulation, and mitochondrial dysfunction. These pathways represent more tractable and biologically grounded targets for future therapeutic exploration in DMD.

This study had several limitations. First, the integrative analysis was based on publicly available datasets from different cohorts and platforms, and part of the study involved cross-species comparisons between human and mouse data, which may have introduced some heterogeneity. Second, this study did not directly assess downstream pathways or *in vivo* therapeutic effects. Future studies should include more detailed functional experiments, and *in vivo* intervention studies to clarify the role of the miR-30c-5p/SOCS3 axis in DMD.

## 5 Conclusion

The miR-30c-5p/SOCS3 axis warrants further mechanistic investigation and may inform the development of novel therapeutic strategies for DMD.

## Data Availability

The original contributions presented in the study are included in the article/Supplementary Material, further inquiries can be directed to the corresponding author. The datasets supporting the findings of this study are publicly available in the GEO database: https://www.ncbi.nlm.nih.gov/geo.

## References

[B1] Amirouche A. Jahnke V. E. Lunde J. A. Koulmann N. Freyssenet D. G. Jasmin B. J. (2017). Muscle-specific microRNA-206 targets multiple components in dystrophic skeletal muscle representing beneficial adaptations. Am. J. Physiol. Cell Physiol. 312, C209–C221. 10.1152/ajpcell.00193.2016 28003225

[B2] Ballarino M. Morlando M. Fatica A. Bozzoni I. (2016). Non-coding RNAs in muscle differentiation and musculoskeletal disease. J. Clin. Invest. 126, 2021–2030. 10.1172/JCI84039 27249675 PMC4887180

[B3] Bhattarai S. Li Q. Ding J. Liang F. Gusev E. Lapohos O. (2022). TLR4 is a regulator of trained immunity in a murine model of Duchenne muscular dystrophy. Nat. Commun. 13, 879. 10.1038/s41467-022-28502-z 35169163 PMC8847425

[B4] Bier A. Berenstein P. Kronfeld N. Morgoulis D. Ziv-Av A. Goldstein H. (2018). Placenta-derived mesenchymal stromal cells and their exosomes exert therapeutic effects in Duchenne muscular dystrophy. Biomaterials 174, 67–78. 10.1016/j.biomaterials.2018.04.055 29783118

[B5] Bin X. Cheng J. Y. Deng Z. Y. Li B. Xu X. H. Liu O. S. (2024). circMTO1/miR-30c-5p/SOCS3 axis alleviates oral submucous fibrosis through inhibiting fibroblast-myofibroblast transition. J. Oral Pathol. Med. 53, 468–479. 10.1111/jop.13524 38802299

[B6] Birnkrant D. J. Bushby K. Bann C. M. Alman B. A. Apkon S. D. Blackwell A. (2018). Diagnosis and management of Duchenne muscular dystrophy, part 2: respiratory, cardiac, bone health, and orthopaedic management. Lancet Neurol. 17, 347–361. 10.1016/S1474-4422(18)30025-5 29395990 PMC5889091

[B7] Boppart M. D. Burkin D. J. Kaufman S. J. (2011). Activation of AKT signaling promotes cell growth and survival in alpha7beta1 integrin-mediated alleviation of muscular dystrophy. Biochim. Biophys. Acta 1812, 439–446. 10.1016/j.bbadis.2011.01.002 21216283 PMC3046458

[B8] Bushby K. M. Hill A. Steele J. G. (1999). Failure of early diagnosis in symptomatic Duchenne muscular dystrophy. Lancet 353, 557–558. 10.1016/S0140-6736(99)01027-2 10028989

[B9] Cardone N. Taglietti V. Baratto S. Kefi K. Periou B. Gitiaux C. (2023). Myopathologic trajectory in Duchenne muscular dystrophy (DMD) reveals lack of regeneration due to senescence in satellite cells. Acta Neuropathol. Commun. 11, 167. 10.1186/s40478-023-01662-8 37858263 PMC10585739

[B10] Ceolotto G. Giannella A. Albiero M. Kuppusamy M. Radu C. Simioni P. (2017). miR-30c-5p regulates macrophage-mediated inflammation and pro-atherosclerosis pathways. Cardiovasc. Res. 113, 1627–1638. 10.1093/cvr/cvx153 29016810

[B11] Chen G. Y. Nunez G. (2010). Sterile inflammation: sensing and reacting to damage. Nat. Rev. Immunol. 10, 826–837. 10.1038/nri2873 21088683 PMC3114424

[B12] Chen Y. Wang X. (2020). miRDB: an online database for prediction of functional microRNA targets. Nucleic Acids Res. 48, D127–D131. 10.1093/nar/gkz757 31504780 PMC6943051

[B13] Chen Y. Gao D. Y. Huang L. (2015). *In vivo* delivery of miRNAs for cancer therapy: challenges and strategies. Adv. Drug Deliv. Rev. 81, 128–141. 10.1016/j.addr.2014.05.009 24859533 PMC5009470

[B14] Chin C. H. Chen S. H. Wu H. H. Ho C. W. Ko M. T. Lin C. Y. (2014). cytoHubba: identifying hub objects and sub-networks from complex interactome. BMC Syst. Biol. 8, S11. 10.1186/1752-0509-8-S4-S11 25521941 PMC4290687

[B15] Das A. Ganesh K. Khanna S. Sen C. K. Roy S. (2014). Engulfment of apoptotic cells by macrophages: a role of microRNA-21 in the resolution of wound inflammation. J. Immunol. 192, 1120–1129. 10.4049/jimmunol.1300639 24391209 PMC4358325

[B16] Diener C. Keller A. Meese E. (2022). Emerging concepts of miRNA therapeutics: from cells to clinic. Trends Genet. 38, 613–626. 10.1016/j.tig.2022.02.006 35303998

[B17] Duan D. Goemans N. Takeda S. Mercuri E. Aartsma-Rus A. (2021). Duchenne muscular dystrophy. Nat. Rev. Dis. Prim. 7, 13. 10.1038/s41572-021-00248-3 33602943 PMC10557455

[B18] Duisters R. F. Tijsen A. J. Schroen B. Leenders J. J. Lentink V. van der Made I. (2009). miR-133 and miR-30 regulate connective tissue growth factor: implications for a role of microRNAs in myocardial matrix remodeling. Circ. Res. 104, 170–178. 10.1161/CIRCRESAHA.108.182543 19096030

[B19] Erriquez D. Perini G. Ferlini A. (2013). Non-coding RNAs in muscle dystrophies. Int. J. Mol. Sci. 14, 19681–19704. 10.3390/ijms141019681 24084719 PMC3821580

[B20] Fernandez-Simon E. Pinol-Jurado P. Gokul-Nath R. Unsworth A. Alonso-Perez J. Schiava M. (2024). Single cell RNA sequencing of human FAPs reveals different functional stages in Duchenne muscular dystrophy. Front. Cell Dev. Biol. 12, 1399319. 10.3389/fcell.2024.1399319 39045456 PMC11264872

[B21] Giordano C. Mojumdar K. Liang F. Lemaire C. Li T. Richardson J. (2015). Toll-like receptor 4 ablation in mdx mice reveals innate immunity as a therapeutic target in Duchenne muscular dystrophy. Hum. Mol. Genet. 24, 2147–2162. 10.1093/hmg/ddu735 25552658 PMC4380065

[B22] Gissel H. (2005). The role of Ca2+ in muscle cell damage. Ann. N. Y. Acad. Sci. 1066, 166–180. 10.1196/annals.1363.013 16533926

[B23] Gou L. T. Zhu Q. Liu M. F. (2023). Small RNAs: an expanding world with therapeutic promises. Fundam. Res. 3, 676–682. 10.1016/j.fmre.2023.04.011 38933305 PMC11197668

[B24] Hu J. Lin F. Yin Y. Shang Y. Xiao Z. Xu W. (2023). Adipocyte-derived exosomal miR-30c-5p promotes ovarian angiogenesis in polycystic ovary syndrome *via* the SOCS3/STAT3/VEGFA pathway. J. Steroid Biochem. Mol. Biol. 230, 106278. 10.1016/j.jsbmb.2023.106278 36870372

[B25] Huang H. Y. Lin Y. C. Cui S. Huang Y. Tang Y. Xu J. (2022). miRTarBase update 2022: an informative resource for experimentally validated miRNA-target interactions. Nucleic Acids Res. 50, D222–D230. 10.1093/nar/gkab1079 34850920 PMC8728135

[B26] Hughes M. C. Ramos S. V. Turnbull P. C. Rebalka I. A. Cao A. Monaco C. M. F. (2019). Early myopathy in Duchenne muscular dystrophy is associated with elevated mitochondrial H(2) O(2) emission during impaired oxidative phosphorylation. J. Cachexia Sarcopenia Muscle 10, 643–661. 10.1002/jcsm.12405 30938481 PMC6596403

[B27] Jorgensen S. B. O'Neill H. M. Sylow L. Honeyman J. Hewitt K. A. Palanivel R. (2013). Deletion of skeletal muscle SOCS3 prevents insulin resistance in obesity. Diabetes 62, 56–64. 10.2337/db12-0443 22961088 PMC3526029

[B28] Khattri R. B. Batra A. White Z. Hammers D. Ryan T. E. Barton E. R. (2024). Comparative lipidomic and metabolomic profiling of mdx and severe mdx-apolipoprotein e-null mice. Skelet. Muscle 14, 36. 10.1186/s13395-024-00342-w 39716324 PMC11664822

[B29] Kielbowski K. Bakinowska E. Procyk G. Zietara M. Pawlik A. (2024). The role of MicroRNA in the pathogenesis of Duchenne muscular dystrophy. Int. J. Mol. Sci. 25, 6108. 10.3390/ijms25116108 38892293 PMC11172814

[B30] Komaki H. (2025). Duchenne muscular dystrophy: evolving therapeutic strategies and multidimensional evaluation approaches. Brain Dev. 47, 104397. 10.1016/j.braindev.2025.104397 40712356

[B31] Lebrun P. Cognard E. Bellon-Paul R. Gontard P. Filloux C. Jehl-Pietri C. (2009). Constitutive expression of suppressor of cytokine signalling-3 in skeletal muscle leads to reduced mobility and overweight in mice. Diabetologia 52, 2201–2212. 10.1007/s00125-009-1456-8 19672574

[B32] Lee R. C. Ambros V. (2001). An extensive class of small RNAs in *Caenorhabditis elegans* . Science 294, 862–864. 10.1126/science.1065329 11679672

[B33] Lee R. C. Feinbaum R. L. Ambros V. (1993). The *C. elegans* heterochronic gene lin-4 encodes small RNAs with antisense complementarity to lin-14. Cell 75, 843–854. 10.1016/0092-8674(93)90529-y 8252621

[B34] Lemos D. R. Babaeijandaghi F. Low M. Chang C. K. Lee S. T. Fiore D. (2015). Nilotinib reduces muscle fibrosis in chronic muscle injury by promoting TNF-mediated apoptosis of fibro/adipogenic progenitors. Nat. Med. 21, 786–794. 10.1038/nm.3869 26053624

[B35] Li J. Donath S. Li Y. Qin D. Prabhakar B. S. Li P. (2010). miR-30 regulates mitochondrial fission through targeting p53 and the dynamin-related protein-1 pathway. PLoS Genet. 6, e1000795. 10.1371/journal.pgen.1000795 20062521 PMC2793031

[B36] Li W. Zheng Y. Zhang W. Wang Z. Xiao J. Yuan Y. (2015). Progression and variation of fatty infiltration of the thigh muscles in Duchenne muscular dystrophy, a muscle magnetic resonance imaging study. Neuromuscul. Disord 25, 375–380. 10.1016/j.nmd.2015.02.003 25701397

[B37] Liu N. Williams A. H. Maxeiner J. M. Bezprozvannaya S. Shelton J. M. Richardson J. A. (2012). microRNA-206 promotes skeletal muscle regeneration and delays progression of Duchenne muscular dystrophy in mice. J. Clin. Invest. 122, 2054–2065. 10.1172/JCI62656 22546853 PMC3366415

[B38] Łoboda A. Chamberlain J. S. Dulak J. (2025). Genetic strategies for therapy of Duchenne muscular dystrophy. Mol. Ther. Nucleic Acids 36, 102759. 10.1016/j.omtn.2025.102759 41328300 PMC12664987

[B39] Mareedu S. Million E. D. Duan D. Babu G. J. (2021). Abnormal calcium handling in Duchenne muscular dystrophy: mechanisms and potential therapies. Front. Physiol. 12, 647010. 10.3389/fphys.2021.647010 33897454 PMC8063049

[B40] Milad N. White Z. Tehrani A. Y. Sellers S. Rossi F. M. V. Bernatchez P. (2017). Increased plasma lipid levels exacerbate muscle pathology in the mdx mouse model of Duchenne muscular dystrophy. Skelet. Muscle 7, 19. 10.1186/s13395-017-0136-1 28899419 PMC5596936

[B41] Moore T. M. Lin A. J. Strumwasser K. R. Cory K. Whitney K. Ho T. (2020). Mitochondrial dysfunction is an early consequence of partial or complete dystrophin loss in mdx mice. Front. Physiol. 11, 690. 10.3389/fphys.2020.00690 32636760 PMC7317021

[B42] Nitahara-Kasahara Y. Kuraoka M. Oda Y. Hayashita-Kinoh H. Takeda S. Okada T. (2021). Enhanced cell survival and therapeutic benefits of IL-10-expressing multipotent mesenchymal stromal cells for muscular dystrophy. Stem Cell Res. Ther. 12, 105. 10.1186/s13287-021-02175-y 33541428 PMC7860619

[B43] Pétrilli V. Dostert C. Muruve D. A. Tschopp J. (2007). The inflammasome: a danger sensing complex triggering innate immunity. Curr. Opin. Immunol. 19, 615–622. 10.1016/j.coi.2007.09.002 17977705

[B44] Samani A. Hightower R. M. Reid A. L. English K. G. Lopez M. A. Doyle J. S. (2022). miR-486 is essential for muscle function and suppresses a dystrophic transcriptome. Life Sci. Alliance 5, e202101215. 10.26508/lsa.202101215 35512829 PMC9087951

[B45] Seyhan A. A. (2024). Trials and tribulations of MicroRNA therapeutics. Int. J. Mol. Sci. 25, 1469. 10.3390/ijms25010620 38338746 PMC10855871

[B46] Shannon P. Markiel A. Ozier O. Baliga N. S. Wang J. T. Ramage D. (2003). Cytoscape: a software environment for integrated models of biomolecular interaction networks. Genome Res. 13, 2498–2504. 10.1101/gr.1239303 14597658 PMC403769

[B47] Soblechero-Martín P. López-Martínez A. de la Puente-Ovejero L. Vallejo-Illarramendi A. Arechavala-Gomeza V. (2021). Utrophin modulator drugs as potential therapies for Duchenne and Becker muscular dystrophies. Neuropathol. Appl. Neurobiol. 47, 711–723. 10.1111/nan.12735 33999469 PMC8518368

[B48] Spassov A. Gredes T. Gedrange T. Pavlovic D. Lupp A. Kunert-Keil C. (2011). Increased oxidative stress in dystrophin deficient (mdx) mice masticatory muscles. Exp. Toxicol. Pathol. 63, 549–552. 10.1016/j.etp.2010.04.006 20471229

[B49] Starosta A. Konieczny P. (2021). Therapeutic aspects of cell signaling and communication in Duchenne muscular dystrophy. Cell. Mol. Life Sci. 78, 4867–4891. 10.1007/s00018-021-03818-w 33825942 PMC8233280

[B50] Steinberg G. R. McAinch A. J. Chen M. B. O'Brien P. E. Dixon J. B. Cameron-Smith D. (2006). The suppressor of cytokine signaling 3 inhibits leptin activation of AMP-kinase in cultured skeletal muscle of obese humans. J. Clin. Endocrinol. Metab. 91, 3592–3597. 10.1210/jc.2006-0565 16822822

[B51] Szklarczyk D. Gable A. L. Nastou K. C. Lyon D. Kirsch R. Pyysalo S. (2021). The STRING database in 2021: customizable protein-protein networks, and functional characterization of user-uploaded gene/measurement sets. Nucleic Acids Res. 49, D605–D712. 10.1093/nar/gkaa1074 33237311 PMC7779004

[B52] Taetzsch T. Shapiro D. Eldosougi R. Myers T. Settlage R. E. Valdez G. (2021). The microRNA miR-133b functions to slow Duchenne muscular dystrophy pathogenesis. J. Physiol. 599, 171–192. 10.1113/JP280036 32991751 PMC8418193

[B53] Trifunov S. Natera-de Benito D. Exposito Escudero J. M. Ortez C. Medina J. Cuadras D. (2020). Longitudinal study of three microRNAs in Duchenne muscular dystrophy and becker muscular dystrophy. Front. Neurol. 11, 304. 10.3389/fneur.2020.00304 32373058 PMC7186470

[B54] Tripodi L. Villa C. Molinaro D. Torrente Y. Farini A. (2021). The immune system in Duchenne muscular dystrophy pathogenesis. Biomedicines 9, 1447. 10.3390/biomedicines9101447 34680564 PMC8533196

[B55] Vincik L. Y. Dautel A. D. Staples A. A. Lauck L. V. Armstrong C. J. Howard J. T. (2024). Evolving role of viltolarsen for treatment of Duchenne muscular dystrophy. Adv. Ther. 41, 1338–1350. 10.1007/s12325-024-02796-w 38376743

[B56] Wang L. Chen X. Wang Y. Zhao L. Zhao X. Wang Y. (2020). MiR-30c-5p mediates the effects of panax notoginseng saponins in myocardial ischemia reperfusion injury by inhibiting oxidative stress-induced cell damage. Biomed. Pharmacother. 125, 109963. 10.1016/j.biopha.2020.109963 32036220

[B57] Wang Y. Chang Y. Zhang P. Zheng Z. Ai X. Zhang S. (2025). Association between triglycerides and remnant cholesterol levels and spine bone mineral density in Duchenne muscular dystrophy. Lipids Health Dis. 24, 209. 10.1186/s12944-025-02302-3 40490739 PMC12147359

[B58] Wightman B. Ha I. Ruvkun G. (1993). Posttranscriptional regulation of the heterochronic gene lin-14 by lin-4 mediates temporal pattern formation in *C. elegans* . Cell 75, 855–862. 10.1016/0092-8674(93)90530-4 8252622

[B59] Wilson D. G. S. Tinker A. Iskratsch T. (2022). The role of the dystrophin glycoprotein complex in muscle cell mechanotransduction. Commun. Biol. 5, 1022. 10.1038/s42003-022-04014-y 36168044 PMC9515174

[B60] Yang Z. Hulver M. McMillan R. P. Cai L. Kershaw E. E. Yu L. (2012). Regulation of insulin and leptin signaling by muscle suppressor of cytokine signaling 3 (SOCS3). PLoS One 7, e47493. 10.1371/journal.pone.0047493 23115649 PMC3480378

[B61] Yedigaryan L. Sampaolesi M. (2021). Therapeutic implications of miRNAs for muscle-wasting conditions. Cells 10, 3035. 10.3390/cells10113035 34831256 PMC8616481

[B62] Zou Y. F. Liao W. T. Fu Z. J. Zhao Q. Chen Y. X. Zhang W. (2017). MicroRNA-30c-5p ameliorates hypoxia-reoxygenation-induced tubular epithelial cell injury via HIF1alpha stabilization by targeting SOCS3. Oncotarget 8, 92801–92814. 10.18632/oncotarget.21588 29190957 PMC5696223

