# Supplementary material for: The miR-30c-5p/SOCS3 axis is a potential driver of inflammation and metabolic imbalance in Duchenne muscular dystrophy
Source: Front Cell Dev Biol. 2026 May 22;14:1841851. doi: 10.3389/fcell.2026.1841851 (PMC13236648; doi:10.3389/fcell.2026.1841851)
Supplement: Supplementary file 2 [file Table1.docx]

SOCS3：

Human： GUUU----------UUUAAUAAUGUUUUACAA-

Mouse： ----------UUUGAUAAUGUUUUACAA-

SOCS3: 5′ ...CUUGUUUUUUAAUAAUGUUUACA ... 3′

miR-30c-5p： 3′ CGACUCUCACAUCCUACAAAUGU 5′
