# Supplementary material for: The miR-30c-5p/SOCS3 axis is a potential driver of inflammation and metabolic imbalance in Duchenne muscular dystrophy
Source: Front Cell Dev Biol. 2026 May 22;14:1841851. doi: 10.3389/fcell.2026.1841851 (PMC13236648; doi:10.3389/fcell.2026.1841851)
Supplement: Supplementary file 3 [file Table3.docx]

**Supplementary Table. Sequences of primers and oligonucleotides used for qRT-PCR and transfection experiments**

| **Gene** | **Primer** | |
| --- | --- | --- |
| U6 | F | CTCGCTTCGGCAGCACATATAC |
|  | R | AACGCTTCACGAATTTGCGT |
| miR-30c-5p | RT | GTCGTATCCAGTGCAGGGTCCGAGGTATTCGCACTGGATACGACAGCTGA |
|  | F | TGTAAACATCCTACACTCTCAGC |
|  | R | AGTGCAGGGTCCGAGGTATT |
| Actin | F | TCCTCCTGAGCGCAAGTACTCC |
|  | R | CATACTCCTGCTTGCTGATCCAC |
| SOCS3 | F | GGACCAAGAACCTACGCATCCA |
|  | R | CACCAGCTTGAGTACACAGTCG |

F: Forward Primers (5’-3’); R: Reverse Primers (5’-3’)

RT: Reverse Transcription primer (5’-3’)

| Oligonucleotide | Sequence (5′–3′) |
| --- | --- |
| miR-30c-5p mimic | UGUAAACAUCCUACACUCUCAGC |
| miR-30c-5p inhibitor | GCUGAGAGUGUAGGAUGUUUACA |
| MicroRNA mimic negative control | UUGUACUACACAAAAGUACUG |
| MicroRNA inhibitor negative control | CAGUACUUUUGUGUAGUACAA |
| si-SOCS3 sense | GGUCACCCACAGCAAGUUUTT |
| si-SOCS3 antisense | AAACUUGCUGUGGGUGACCTT |

| Construct | Species | Gene | Reference sequence | Vector |
| --- | --- | --- | --- | --- |
| SOCS3 overexpression plasmid | Mouse | SOCS3 | NM_007707.4 | pcDNA3.1(+) |
